# Insurance Coverage and Distribution of DXA Screening in Saudi Arabia: Evidence from Major Healthcare Settings

**DOI:** 10.3390/healthcare14142218

**Published:** 2026-07-21

**Authors:** Naof Saleem Al-Ansary, Adnan Matouk Almarzouq

**Affiliations:** 1 Department of Public Health, College of Public Health, Imam Abdulrahman Bin Faisal University, Dammam 34212, Saudi Arabia; 2 Department of Internal Medicine, Dr. Sulaiman Al Habib Hospital-Al Khobar, Al Khobar 34423, Saudi Arabia; adnan.almarzouq@drsulaimanalhabib.com

**Keywords:** insurance coverage, osteoporosis, dual-energy X-ray absorptiometry, detection, Saudi Arabia

## Abstract

**Highlights:**

**What are the main findings?**

- Differences in healthcare organization and insurance financing were observed in the distribution of patients receiving DXA examinations.
- Self-pay patients were predominantly represented in public healthcare facilities.

**What are the implications of the main implications of the main findings?**

- The distribution of patients receiving DXA examinations differs significantly by insurance type.
- Strengthening equitable insurance coverage and integrated healthcare delivery may support more consistent osteoporosis diagnostic services.

**Abstract:**

**Background:** Osteoporosis is a major public health concern, and early detection through dual-energy X-ray absorptiometry (DXA) has been pivotal. However, evidence on how insurance coverage relates to the distribution of patients receiving DXA examinations in Saudi Arabia remains limited. This study examines the distribution of patients receiving DXA examinations across different insurance types and healthcare settings among adult patients in Saudi Arabia. **Methods:** This retrospective observational study used de-identified electronic health record data from a public tertiary academic center and a large private healthcare system in Saudi Arabia between 1 January 2023 and 28 February 2026. Dual-energy X-ray absorptiometry (DXA) was employed as an imaging method used to measure bone mineral density and support osteoporosis diagnosis and fracture-risk assessment. Adult patients aged ≥18 years with completed DXA examinations recorded in radiology service records were included. This study assessed the distribution of patients receiving DXA examinations across insurance types, healthcare setting, ordering department, and year of service. Insurance type was categorized as government coverage, private insurance, or self-pay. Descriptive statistics, chi-square tests, one-way ANOVA, Cramér’s V, and standardized residuals were used to compare distribution patterns across groups. **Results:** The data of 8930 DXA recipients were analyzed. Clear differences were observed in the distribution of patients by insurance type and healthcare setting (*p* < 0.001). Privately insured patients were predominantly treated in private facilities, whereas government-insured and self-pay patients were primarily concentrated in public healthcare facilities. Significant variations were also observed across healthcare departments and over time, demonstrating strong system-level and financial stratification in the distribution of patients receiving DXA examinations. **Conclusions:** The findings of this study demonstrate significant differences in the distribution of patients receiving DXA examinations according to insurance type and healthcare setting. Notably, self-pay patients were predominantly managed in public healthcare facilities, whereas privately insured patients primarily received DXA examinations in private healthcare settings. These observed patterns suggest that differences in healthcare organization and insurance financing may be associated with where patients receive DXA examinations; however, because this study included only individuals who underwent DXA examinations, the findings should not be interpreted as measures of screening uptake, access, or equity among all patients eligible for osteoporosis screening. Future healthcare strategies should focus on strengthening equitable insurance coverage, improving coordination between public and private healthcare sectors, standardizing referral pathways, and supporting integrated preventive care in line with Saudi Arabia’s Vision 2030 healthcare transformation.

## 1. Introduction

Osteoporosis is a systemic skeletal disorder characterized by reduced bone mass and deterioration of the bone microarchitecture, leading to an increased risk of fractures and substantial global morbidity, mortality, and healthcare costs [1]. Dual-energy X-ray absorptiometry (DXA) is the gold standard for assessing bone mineral density and plays a central role in identifying individuals at high fracture risk, enabling timely preventive and therapeutic interventions [2]. DXA is a non-invasive imaging technique that provides quantitative measurements of bone mineral density and is routinely used for the diagnosis, risk stratification, and clinical management of osteoporosis. In the present study, DXA examination records from participating in public and private healthcare settings were used to identify the study population, comprising adult patients (≥18 years) who underwent complete DXA examinations during the study period. Accordingly, this study evaluates the distribution of patients who received DXA examinations across different insurance categories and healthcare settings rather than estimating population-level screening uptake among all individuals eligible for osteoporosis screening. Despite well-established screening recommendations, substantial gaps in osteoporosis care persist, with many eligible individuals not undergoing DXA screening or receiving appropriate follow-up, reflecting ongoing deficiencies in preventive service delivery [1].

Access to preventive healthcare services, including screening for chronic conditions and preventive measures, has been closely associated with health insurance coverage as a key structural determinant. Evidence from the United States indicates that older adults with comprehensive insurance coverage are significantly more likely to undergo osteoporosis screening compared to those with limited or no coverage [3]. Similar associations between insurance status and preventive service use have been reported across a range of preventive interventions and healthcare settings, highlighting the pivotal role that systemic factors play in shaping preventive care delivery [4,5,6].

In Saudi Arabia, demographic changes and rising chronic disease incidence rates exacerbate the public health concern towards osteoporosis. Saudi osteoporosis guidelines recommend DXA screening for women and men aged 60 years and older, as well as for younger individuals with known risk factors [7,8]. However, real-world data establishing associations between healthcare settings, insurance status, and the distribution of patients receiving DXA examinations remain limited. Available local studies suggest variability in clinical practice related to osteoporosis screening, shedding light on system-level and structural variables that may influence the utilization of recommended preventive service guidelines [9,10]. To date, the distribution of patients receiving DXA examinations across insurance coverage types and service delivery settings in Saudi Arabia has not been well described.

Outlining the pattern of distribution of patients receiving DXA examinations across insurance coverage types and healthcare settings is particularly relevant in the context of the ongoing healthcare reform and the establishment of universal health insurance schemes under the country’s comprehensive reform movement, Saudi Vision 2030. This study aims to retrospectively analyze electronic health record (EHR) data to examine the distribution of adult patients receiving DXA examinations in Saudi Arabia. Specifically, we assess the proportion of completed examinations segmented by insurance type and healthcare setting. Real-world distribution patterns of patients receiving DXA examinations may help identify potential differences across healthcare settings and insurance coverage types [6,11].

## 2. Materials and Methods

This retrospective observational study was conducted using de-identified electronic health record (EHR) data from two major healthcare settings in Saudi Arabia: a public tertiary academic center and a large private healthcare system. The study period spanned from 1 January 2023 to 28 February 2026. Data for 2026 represent a partial calendar year and include only DXA examinations performed between 1 January and 28 February 2026. A retrospective design was used to assess real-world patterns and distribution of DXA service receipts using EHR data [11].

The study was conducted on data of adult patients (≥18 years) who underwent dual-energy X-ray absorptiometry (DXA) examinations during the study period. DXA scans were identified using radiology service records and procedure codes for bone mineral density assessment of the lumbar spine and/or hip [7]. The study population comprised adult patients (≥18 years) who underwent complete DXA examinations during the study period. Eligible participants were identified from radiology service records within the participating public and private healthcare institutions using standardized procedure codes for bone mineral density assessment. Because this retrospective study was designed to examine the distribution of patients who received DXA examinations, the sampling frame was restricted to individuals with completed DXA records available in the electronic health record database. Consequently, the analysis describes the distribution of patients receiving DXA examinations across insurance types and healthcare settings rather than estimating screening uptake among all patients eligible for osteoporosis screening.

Health insurance coverage and type of healthcare provider were the primary variables of interest. The EHR registration and billing data were utilized to extract patients’ insurance status, which was categorized into three groups: national coverage, private insurance, and self-pay. The insurance type is assigned in the EHR at the time DXA service is provided and reflects the patient’s coverage status at the time of the encounter.

For this study, the distribution of patients receiving DXA examinations was defined as the proportional distribution of adult patients who underwent completed DXA examinations across the insurance categories and healthcare settings included in the study. Healthcare setting referred to the participating public and private healthcare institutions. Structural determinants of access were defined as characteristics of healthcare organization and service delivery, whereas financial determinants referred to differences in insurance coverage and payment mechanisms that may influence where patients received DXA examinations.

The study outcome was the distribution of patients receiving DXA examinations across insurance types and healthcare settings. Since all included patients had completed DXA examinations, the analysis focused on describing and comparing distribution patterns rather than screening uptake or completion rates.

Additional variables included age (years), sex (male/female), nationality (Saudi/non-Saudi), healthcare setting (public or private), ordering department group (Primary Care, Endocrine, Musculoskeletal, Geriatrics, Radiology, and Other Clinical Specialties), and year of service (2023–2026). These variables were included to describe the study population and to assess differences in the distribution of patients receiving DXA examinations across healthcare settings and demographic variables.

Descriptive statistics were used to summarize patient characteristics, stratified by insurance type and healthcare setting. Categorical variables were presented as frequencies and percentages, while continuous variables were summarized using means and standard deviations. Percentages for categorical variables were calculated using column proportions.

Comparisons across insurance groups (national coverage, private insurance plan, and self-pay) were performed using the chi-square test for categorical variables and one-way analysis of variance (ANOVA) for continuous variables. Normality and homogeneity of variance assumptions were assessed prior to ANOVA. All analyses were two-tailed, and a *p*-value of <0.05 was considered the threshold for statistical significance. Statistical analyses were performed using IBM SPSS Statistics version 31 (IBM Corp., Armonk, NY, USA).

## 3. Results

A total of 8930 individuals who underwent DXA screening were included in the analysis, stratified by insurance type into government (n = 841), private (n = 5789), and self-pay (n = 2300) groups (Table 1).

### 3.1. Baseline Characteristics by Insurance Type

The mean age of participants was similar across insurance groups, with a small but statistically significant difference (government: 59.65 ± 16.18 years; private: 60.58 ± 13.52 years; self-pay: 59.54 ± 15.42 years; *p* = 0.006). Females constituted most DXA recipients across all insurance types; however, proportions varied (*p* < 0.001), with a higher proportion of males observed in the private insurance group (25.8%) compared to government (18.4%) and self-pay groups (16.5%). Most patients were Saudi across all groups, with higher representation in the private insurance group (89.9%) compared to government (83.5%) and self-pay groups (83.7%) (*p* < 0.001).

Marked differences in healthcare settings were observed across insurance types (all *p* < 0.001). Patients covered by government insurance were exclusively managed in public healthcare facilities, whereas nearly all privately insured patients received DXA examinations in private healthcare facilities. In contrast, self-pay patients were predominantly managed in public healthcare facilities (90.6%). Patterns varied significantly in the distribution of DXA examinations by year, with observed concentrations in 2025 and 2026 of publicly insured patients, while private and self-pay patients were more evenly distributed across earlier years, particularly 2024.

Referral patterns also differed across insurance types. Radiology accounted for most referrals in the public (68.1%) and self-pay groups (86.1%), whereas primary care was the leading source of referring among privately insured patients (52.3%). Musculoskeletal and endocrine specialties contributed more prominently within the private insurance group.

### 3.2. Characteristics by Healthcare Setting

Of the total cohort, 2996 (33.5%) patients were treated in public facilities and 5934 (66.5%) in private facilities (Table 2). Patients in private settings were slightly older than those in public settings (60.61 ± 13.60 vs. 59.47 ± 15.56 years, *p* < 0.001). The proportion of males was higher in private facilities (26.1%) compared to public facilities (16.0%), while females predominated in both settings (*p* < 0.001). Saudi nationals were more frequently represented in private facilities (89.6%), whereas non-Saudis were more commonly seen in public settings.

Strong differences in healthcare system characteristics were identified between settings (all *p* < 0.001). Government insurance was confined to public facilities, while private insurance was overwhelmingly concentrated in private settings. Self-pay patients predominantly sought care in public facilities. Referring departments also varied substantially across healthcare settings (Table 3). Radiology accounted for most DXA orders in public facilities (86.9%), while primary care was the dominant referring source in private facilities (52.8%). Musculoskeletal and endocrine specialties were more prominent in private settings, whereas geriatrics referrals were almost exclusively observed in public care.

Temporal patterns also differed between settings, with public facilities contributing a higher proportion of examinations in 2023 and 2026, whereas private facilities accounted for most examinations in 2024 and 2025 (Table 4).

### 3.3. Association Between Insurance Type and Healthcare Structure

A strong association was identified between healthcare setting and insurance type (χ^2^ (2) = 7737.55, *p* < 0.001), with a large effect size (Cramér’s V = 0.66). Government insurance was overrepresented in public facilities and virtually absent in private settings, while private insurance was highly concentrated in private facilities. Self-pay patients were disproportionately managed in public facilities.

Similarly, robust associations were observed between ordering departments and insurance type (χ^2^ (10) = 6872.52, *p* < 0.001; Cramér’s V = 0.62). Primary care and musculoskeletal services were strongly associated with private insurance, whereas radiology showed overrepresentation among self-paying patients. Geriatrics was predominantly associated with government insurance.

### 3.4. Temporal Trends in Insurance Type and Healthcare Setting

A significant association was identified between year of service and insurance type (χ^2^ (6) = 2979.72, *p* < 0.001), with a moderate-to-strong effect size (Cramér’s V = 0.41). In 2023, the distribution of patients receiving DXA examinations was largely driven by self-pay patients, with minimal contribution from government insurance. In 2024, private insurance became dominant, while self-pay remained substantial. By 2025, the distribution of patients receiving DXA examinations shifted toward government insurance, accompanied by a decline in self-pay patients. In 2026, government insurance predominated; however, these findings are based on data collected only between 1 January and 28 February 2026. Therefore, the observed distribution should be interpreted cautiously because it reflects a partial-year dataset with a substantially smaller sample size than previous years.

A weaker but statistically significant association was also observed between year and healthcare setting (χ^2^ (3) = 577.71, *p* < 0.001; Cramér’s V = 0.18). The distribution of patients receiving DXA examinations was relatively balanced between healthcare settings in 2023, followed by a predominance of private healthcare facilities in 2024 and 2025 and a reversal toward public healthcare facilities in 2026.

## 4. Discussion

This study examined the distribution of patients receiving DXA examinations across insurance types and healthcare settings in Saudi Arabia using a large real-world dataset. The findings demonstrated significant differences in the distribution of patients receiving DXA examinations according to insurance type and healthcare setting. Specifically, privately insured patients were predominantly managed within private healthcare settings, whereas government-insured and self-pay patients were largely concentrated in public healthcare facilities. In addition, substantial variation was observed across clinical departments and over time, indicating that healthcare organization and insurance financing were associated with differences in the distribution of patients receiving DXA examinations.

Significant differences in the distribution of patients receiving DXA examinations were observed according to insurance type and healthcare setting. National coverage was exclusively represented within public healthcare facilities, whereas private insurance was predominantly associated with private healthcare institutions. Similarly, self-pay patients were disproportionately represented in public healthcare facilities. These findings reflect a close relationship between insurance type and healthcare setting within the study population and are consistent with previous evidence suggesting limited integration between public and private healthcare financing and service delivery systems [5,7,9,10]. However, because the present study was descriptive in nature, these findings should be interpreted as observed distribution patterns rather than evidence of causal mechanisms or differences in healthcare access.

The observed distribution of DXA recipients also highlights the presence of both system-level and financial stratification in the delivery of osteoporosis diagnostic services. System-level stratification refers to differences in healthcare organization, referral pathways, and service delivery between public and private healthcare sectors, whereas financial stratification relates to differences in insurance coverage and payment mechanisms that may influence where patients receive DXA examinations. Previous studies have reported that fragmented healthcare systems and variations in insurance financing are associated with differences in the delivery of preventive healthcare services by directing patients toward specific healthcare providers and diagnostic pathways [5,12,13]. Consistent with this evidence, the present study found that the distribution of patients receiving DXA examinations differed substantially according to healthcare setting and insurance type. However, because the study was not designed to evaluate individual-level access, screening uptake, or barriers to care, these findings should be interpreted as describing the observed distribution of DXA recipients rather than directly demonstrating differences in healthcare access.

Self-pay patients were disproportionately represented in public healthcare facilities. This observed distribution is consistent with previous studies reporting that healthcare financing arrangements and insurance coverage are associated with patterns of preventive healthcare utilization [4,13,14,15]. In Saudi Arabia, public healthcare institutions continue to play a central role in delivering subsidized healthcare services to eligible residents, whereas access to private healthcare is more closely linked to private insurance coverage or direct out-of-pocket payment [10,13,15,16]. Nevertheless, because the present study did not examine patient-level reasons for healthcare facility selection, it cannot determine whether this pattern reflects financial considerations, institutional referral practices, patient preferences, or other organizational factors. Therefore, the findings should be interpreted as describing the observed distribution of DXA recipients rather than demonstrating the mechanisms responsible for healthcare facility selection. Further research is warranted to investigate the factors contributing to this observed distribution.

Differences in clinical care pathways and practice guidelines are further highlighted by the substantial correlation between insurance type and ordering department. While radiology dominated referrals in public facilities, especially among self-paying patients, primary care and musculoskeletal services were the main drivers of DXA referrals in private settings. These differences may reflect variations in care coordination models, referral practices, and the role of primary care across healthcare sectors, which may contribute to differences in referral patterns [17,18,19,20]. Previous research has highlighted the critical role that primary care plays in promoting osteoporosis detection and preventive screening, indicating that increased primary care engagement may enable more organized and systematic screening pathways [21,22,23,24,25].

Significant temporal variations in the distribution of patients receiving DXA examinations were also observed. In contrast to subsequent years, especially 2025 and 2026, which showed a significant increase in patients with government insurance, the early years (2023–2024) were defined by a greater reliance on self-pay and private insurance. The contemporary changes in the health system, accompanied by the expanded national insurance schemes driven by Saudi Vision 2030, may be reflected in the temporal variance in distribution of patients receiving DXA examinations [16,17,26]. Dynamic system-level changes, consistent with the healthcare transformation, are seen in distribution shifts observed in healthcare settings with a rise in private sector consumption in 2024–2025 and a reversal toward public institutions in 2026 [16,17]. However, the 2026 findings are based on data collected only during January and February and therefore represent a partial calendar year. Consequently, the observed temporal patterns should be interpreted cautiously because the shorter observation period and smaller sample size may not fully represent annual trends.

The findings of this study are consistent with previous research demonstrating that insurance coverage is associated with the delivery and use of preventive healthcare services [24]. Individuals with more comprehensive insurance coverage are more likely to undergo osteoporosis screening and other preventive interventions [3,4]. Ongoing disparities in healthcare service utilization across socioeconomic and insurance layers have also been extensively reported.

Recent national guidelines in Saudi Arabia stress the need for osteoporosis screening and early diagnosis [7,21]. However, research has shown that there are gaps in DXA service awareness, access, and consumption, especially among specific populations [9,25]. The current study adds to this body of literature by offering comprehensive, system-level evidence describing how the distribution of patients receiving DXA examinations varies according to insurance type and healthcare setting.

The results of this study have significant implications for service delivery and healthcare policy. The substantial correlation between insurance type and healthcare setting indicates that there is still a lack of cross-sector integration, which could jeopardize equity in access to preventive care [5,12,13]. These findings further highlight the importance of equitable insurance coverage and integrated healthcare delivery. Previous studies have shown that comprehensive insurance coverage reduces financial barriers to preventive healthcare services and promotes more equitable access to diagnostic examinations [4,6,15]. Likewise, greater integration between public and private healthcare systems can improve continuity of care, reduce fragmentation, and facilitate more consistent delivery of preventive services [12,13,16]. In the context of Saudi Arabia’s Vision 2030 healthcare reforms, strengthening insurance coverage and coordination between healthcare sectors may contribute to a more consistent and equitable distribution of osteoporosis diagnostic services across healthcare settings. Furthermore, enhancing integration between the public and private healthcare systems may reduce fragmentation, strengthen the continuity of care, and support more consistent delivery of osteoporosis diagnostic services, including DXA examinations.

The prevalence of self-paying patients in public facilities emphasizes the need to reinforce care dynamics and establish insurance coverage standards that alleviate financial obstacles. Evidence has consistently shown that policies expanding insurance enrollment and preventive care coverage are associated with improved delivery of preventive healthcare services and better health outcomes [4,6,15,27].

Moreover, there is an opportunity to strengthen primary care-led screening pathways, particularly in public settings, given the variability in referral practices across departments. Expanding the role of primary care in osteoporosis screening could support earlier identification of osteoporosis through more consistent diagnostic pathways [2,23,25,28,29].

This study has several strengths. It leveraged a large, multi-year dataset derived from electronic health records, empowering a comprehensive assessment of real-world patterns of distribution of patients receiving DXA examinations [11]. The findings are more applicable throughout the Saudi healthcare system when both public and private healthcare settings are included. Furthermore, the use of effect size measurements and standardized residuals offers a reliable evaluation of correlations that goes beyond traditional significance tests.

Although the present descriptive analyses identified significant differences distribution of patients receiving DXA examinations across insurance types and healthcare settings, the findings should be interpreted in the context of the study design. Multivariable regression models adjusting for demographic and healthcare system characteristics could provide additional insight into the independent association between insurance type and distribution of patients receiving DXA examinations while accounting for potential confounding factors. Several limitations should be acknowledged. First, only those who had DXA exams were included in the study; as a result, those who were eligible but not screened were not included. Consequently, the findings may understate the actual population-level unmet demand for osteoporosis screening since they represent the distribution of patients receiving DXA examinations rather than total screening adoption [1,17]. Because the denominator of individuals eligible for osteoporosis screening was unavailable, this study could not determine screening uptake, utilization rates, or healthcare access across insurance groups. Therefore, the findings should be interpreted as describing the distribution of patients who underwent DXA examinations rather than population-level screening performance.

Second, the analyses were primarily descriptive and did not include multivariable regression models to simultaneously adjust for demographic and healthcare system characteristics, including age, sex, nationality, year of service, healthcare setting, and referring department. Therefore, this study was not designed to evaluate the independent contribution of these variables after adjustment for potential confounding factors. Future studies employing multivariable analytical approaches are warranted to further investigate the independent associations between insurance type and the distribution of patients receiving DXA examinations while accounting for potential confounding variables [11].

Third, the 2026 dataset includes only examinations performed between 1 January and 28 February 2026. As these data represent a partial calendar year, temporal comparisons involving 2026 should be interpreted cautiously because the shorter observation period may not accurately reflect annual patterns.

Lastly, because this study was limited to a few healthcare institutions, the findings may not be fully generalizable to all healthcare settings across Saudi Arabia.

## 5. Conclusions

This study demonstrates that the distribution of patients receiving DXA examinations in Saudi Arabia differs significantly according to insurance type and healthcare setting. Adult patients covered by private insurance predominantly received DXA examinations within private healthcare facilities, whereas government-insured and self-pay patients were primarily managed in public healthcare institutions. The observed distribution also highlights differences related to healthcare organization and insurance financing, indicating the presence of system-level and financial stratification in the distribution of patients receiving DXA examinations.

These findings have important implications for healthcare policy. Strategies to strengthen equitable insurance coverage, reduce financial barriers to preventive healthcare services, improve coordination between public and private healthcare providers, strengthen referral pathways, promote standardized clinical guidelines, and support integrated preventive care should be prioritized in line with Saudi Arabia’s Vision 2030 healthcare transformation [30]. Policymakers should also consider improving interoperability between healthcare sectors to facilitate the continuity of care and promote a more consistent distribution of osteoporosis diagnostic services across healthcare settings. Because this study describes the distribution of patients who underwent DXA examinations rather than screening uptake among all eligible individuals, future research incorporating the population eligible for osteoporosis screening is warranted to evaluate screening uptake, healthcare access, and equity across insurance groups.

## Figures and Tables

**Table 1:** Baseline Characteristics of DXA Recipients by Insurance Type (N = 8930).

Variable	Government (n = 841)	Private (n = 5789)	Self-Pay (n = 2300)	*p*-Value
***Patient Demographics***				
**Age (years), mean ± SD**	59.65 ± 16.18	60.58 ± 13.52	59.54 ± 15.42	0.006
**Gender, n (%)**				<0.001
Male	155 (18.4)	1494 (25.8)	379 (16.5)	
Female	686 (81.6)	4295 (74.2)	1921 (83.5)	
**Nationality, n (%)**				<0.001
Saudi	702 (83.5)	5202 (89.9)	1924 (83.7)	
Non-Saudi	139 (16.5)	587 (10.1)	376 (16.3)	
***Healthcare System Characteristics***				
**Healthcare Setting, n (%)**				<0.001
Governmental	841 (100)	71 (1.2)	2084 (90.6)	
Private	0 (0.0)	5718 (98.8)	216 (9.4)	
**Year of DXA, n (%)**				<0.001
2023	2 (0.2)	595 (10.3)	669 (29.1)	
2024	10 (1.2)	2672 (46.2)	1302 (56.6)	
2025	675 (80.3)	2511 (43.4)	316 (13.7)	
2026	154 (18.3)	11 (0.2)	13 (0.6)	
**Ordering Department Group, n (%)**				<0.001
Primary Care	88 (10.5)	3026 (52.3)	132 (5.7)	
Endocrine	69 (8.2)	862 (14.9)	85 (3.7)	
Musculoskeletal	24 (2.9)	1300 (22.5)	66 (2.9)	
Geriatrics	50 (5.9)	6 (0.1)	5 (0.2)	
Radiology	573 (68.1)	74 (1.3)	1981 (86.1)	
Other Clinical Specialties	37 (4.4)	521 (9.0)	31 (1.3)	

Data are presented as mean ± standard deviation or number (percentage), as appropriate. Percentages are calculated within insurance groups. Comparisons between groups were performed using one-way analysis of variance (ANOVA) for continuous variables and chi-square test for categorical variables. Statistical significance was defined as *p* < 0.05.

**Table 2:** Characteristics of DXA Recipients by Healthcare Setting.

Variable	Public (n = 2996)	Private (n = 5934)	*p*-Value
***Patient Demographics***			
**Age (years), mean ± SD**	59.47 ± 15.56	60.61 ± 13.60	<0.001
**Gender, n (%)**			<0.001
Male	480 (16.0)	1548 (26.1)	
Female	2516 (84.0)	4386 (73.9)	
**Nationality, n (%)**			<0.001
Saudi	2510 (83.8)	5318 (89.6)	
Non-Saudi	486 (16.2)	616 (10.4)	
***Healthcare System Characteristics***			
**Insurance Type, n (%)**			<0.001
Government	841 (28.1)	0 (0.0)	
Private	71 (2.4)	5718 (96.4)	
Self-pay	2084 (69.6)	216 (3.6)	
**Year of DXA, n (%)**			<0.001
2023	645 (21.5)	621 (10.5)	
2024	1196 (39.9)	2788 (47.0)	
2025	980 (32.7)	2522 (42.5)	
2026	175 (5.8)	3 (0.1)	
**Department Group, n (%)**			<0.001
Primary Care	113 (3.8)	3133 (52.8)	
Endocrine	129 (4.3)	887 (14.9)	
Musculoskeletal	42 (1.4)	1348 (22.7)	
Geriatrics	57 (1.9)	4 (0.1)	
Radiology	2604 (86.9)	24 (0.4)	
Other Clinical Specialties	51 (1.7)	538 (9.1)	

Data are presented as mean ± standard deviation or number (percentage), as appropriate. Percentages are calculated within healthcare setting. Comparisons between groups were performed using independent *t*-test for continuous variables and chi-square test for categorical variables. Statistical significance was defined as *p* < 0.05.

**Table 3:** Distribution of Insurance Type by Healthcare Setting and Department Group with Effect Size and Standardized Residuals (N = 8930).

	Government Insurance n (%)	Private Insurance n (%)	Self-Pay n (%)	Total n (%)
**Healthcare Setting**				
Public	841 (28.1) (+18.9)	71 (2.4) (−25.4)	2084 (69.6) (+14.7)	2996 (100)
Private	0 (0.0) (−22.1)	5718 (96.4) (+27.8)	216 (3.6) (−16.3)	5934 (100)
Overall Total	841 (9.4)	5789 (64.8)	2300 (25.8)	8930 (100)
**Department Group**				
Primary Care	88 (2.7) (−12.5)	3026 (93.2) (+19.4)	132 (4.1) (−8.2)	3246 (100)
Endocrine	69 (6.8) (−6.1)	862 (84.8) (+11.3)	85 (8.4) (−3.4)	1016 (100)
Musculoskeletal	24 (1.7) (−10.2)	1300 (93.5) (+17.6)	66 (4.7) (−7.1)	1390 (100)
Geriatrics	50 (82.0) (+25.6)	6 (9.8) (−9.8)	5 (8.2) (−5.9)	61 (100)
Radiology	573 (21.8) (+9.8)	74 (2.8) (−21.7)	1981 (75.4) (+28.3)	2628 (100)
Other Clinical Specialties	37 (6.3) (−4.3)	521 (88.5) (+8.7)	31 (5.3) (−2.9)	589 (100)
Overall Total	841 (9.4)	5789 (64.8)	2300 (25.8)	8930 (100)

Data are presented as number (percentage). Percentages are calculated within row categories. Standardized residuals (in parentheses) indicate deviation from expected counts under independence. Values > |1.96| indicate statistically significant over- or underrepresentation. Associations were assessed using Pearson’s chi-square test. Effect size was measured using Cramér’s V. Effect sizes: Healthcare Setting × Insurance Type: χ^2^ (2) = 7737.55, *p* < 0.001, Cramér’s V = 0.66 Department Group × Insurance Type: χ^2^ (10) = 6872.52, *p* < 0.001, Cramér’s V = 0.62 Standardized residuals are presented as adjusted Pearson residuals (z-scores). Statistical significance was defined as *p* < 0.05.

**Table 4:** Temporal Distribution of DXA Recipients by Insurance Type and Healthcare Setting (2023–2026, N = 8930).

**Year × Insurance Type**	**Government Insurance n (%)**	**Private Insurance n (%)**	**Self-Pay n (%)**	**Total n (%)**
2023	2 (0.2) (−8.9)	595 (47.0) (−6.4)	669 (52.8) (+12.1)	1266 (100)
2024	10 (0.3) (−10.7)	2672 (67.1) (+4.8)	1302 (32.7) (+2.9)	3984 (100)
2025	675 (19.3) (+14.6)	2511 (71.7) (+6.2)	316 (9.0) (−11.4)	3502 (100)
2026	154 (86.5) (+22.8)	11 (6.2) (−21.7)	13 (7.3) (−18.9)	178 (100)
**Year × Healthcare Setting**	**Public Hospital n (%)**	**Private Hospital n (%)**		**Total n (%)**
2023	645 (50.9) (+3.8)	621 (49.1) (−3.8)		1266 (100)
2024	1196 (30.0) (−4.9)	2788 (70.0) (+4.9)		3984 (100)
2025	980 (28.0) (−6.1)	2522 (72.0) (+6.1)		3502 (100)
2026	175 (98.3) (+18.4)	3 (1.7) (−18.4)		178 (100)

Data are presented as number (percentage). Percentages are calculated each year. Standardized residuals indicate deviation from expected frequencies under independence. Values > |1.96| indicate statistically significant over- or underrepresentation. Associations were assessed using Pearson’s chi-square test. Effect size was measured using Cramér’s V. Effect sizes: Year × Insurance Type: χ^2^ (6) = 2979.72, *p* < 0.001, Cramér’s V = 0.41 Year × Healthcare Setting: χ^2^ (3) = 577.71, *p* < 0.001, Cramér’s V = 0.18 Note: Data for 2026 represent a partial calendar year (1 January to 28 February 2026) and should be interpreted with caution because of the shorter observation period and smaller sample size. Statistical significance was defined as *p* < 0.05.

## Data Availability

The datasets presented in this article are not readily available due to the nature of the research and ethical restrictions, supporting data are not available.

## Abbreviations

The following abbreviations are used in this manuscript:

DXA	Dual-energy X-ray absorptiometry
EHR	Electronic health record
IRB	Institutional Review Board
ANOVA	Analysis of variance
